# On Submitting to the *JACMP*

**DOI:** 10.1120/jacmp.v15i5.5249

**Published:** 2014-09-08

This editorial focuses on the common mistakes authors make when preparing a submission for the *JACMP*. In the publishing community, there are few absolute standards, and widely differing guidelines for marking up an article for publication. An article prepared properly for another journal might not be acceptable for the *JACMP* for any number of reasons. I urge you to consult the *JACMP Submission Guidelines* before submitting your article so you can avoid the lost time of having the article declined and having to make changes for a resubmission. Here are a few of the things I find are common errors:

- Submitting the document in A4 format — Our standard at the *JACMP* is US letter format. If the document is submitted in A4 format, it will be changed to US letter; if the article has multiple sections, each section will need to be changed.
- Improper file type for the article — The *JACMP* cannot accept .pdf or Tex documents. Only Word or RTF documents can be accepted.
- Including author/institution information — Our review model at the *JACMP* is double‐blind. I review each article to see if any author or institution identification is included. I make every attempt to redact such information to preserve the anonymity of the review process. We do not need or want a Title Page for your article until after it is accepted and ready for copyediting.
- Improper submission of images and tables — All images and tables must be included in the body of the article. Do not imbed Excel tables in your Word or RTF submission. Instead, use the table creation features of your application to create tables. You need to provide high‐quality figures for your layout editor. These should be in .jpg, .gif, .png or another allowed image file format. Please do not provide figures imbedded in .ppt, .xls, .pdf or .doc files. If the reviewers have access to all the files and tables in the review version article, it is usually not necessary to make them available to the reviewers unless, for some reason, very high‐quality graphics are required to complete a proper review.
- Quality of composition — If English is not your first language, have an individual whose first language is English review your article. This will increase your chances the article will be clear to the reviewers, and also benefit its chances of being accepted for publication.
- Acknowledgements — It is not necessary to include Acknowledgements until after your article is accepted for publication and ready for copyediting. Such acknowledgements often contain information that could make it possible for a reviewer to identify the author or institution.

Thanks to all of our authors for making the *JACMP* your journal of choice for your academic contributions. I hope this editorial will help you as you prepare future submissions.

Michael D. Mills

Editor‐in‐Chief

